# Segmentation-based detection of allelic imbalance and loss-of-heterozygosity in cancer cells using whole genome SNP arrays

**DOI:** 10.1186/gb-2008-9-9-r136

**Published:** 2008-09-16

**Authors:** Johan Staaf, David Lindgren, Johan Vallon-Christersson, Anders Isaksson, Hanna Göransson, Gunnar Juliusson, Richard Rosenquist, Mattias Höglund, Åke Borg, Markus Ringnér

**Affiliations:** 1 Department of Oncology, Clinical Sciences, Lund University, SE-22185 Lund, Sweden; 2 CREATE Health Strategic Centre for Clinical Cancer Research, Lund University, SE-22184 Lund, Sweden; 3 Department of Medical Sciences, Cancer Pharmacology and Informatics, Uppsala University, SE-75185 Uppsala, Sweden; 4 Lund Strategic Research Center for Stem Cell Biology and Cell Therapy, Lund University, SE-22184 Lund, Sweden; 5 Department of Genetics and Pathology, Uppsala University, SE-75185 Uppsala, Sweden

## Abstract

We present a strategy for detection of loss-of-heterozygosity and allelic imbalance in cancer cells from whole genome single nucleotide polymorphism genotyping data. Using a dilution series of a tumor cell line mixed with its paired normal cell line and data generated on Affymetrix and Illumina platforms, including paired tumor-normal samples and tumors characterized by fluorescent *in situ *hybridization, we demonstrate a high sensitivity and specificity of the strategy for detecting both minute and gross allelic imbalances in heterogeneous tumor samples.

## Abstract 2

A strategy is presented for detection of loss-of-heterozygosity and allelic imbalance in cancer cells from whole genome SNP genotyping data.

## Background

Cancer development involves genomic aberrations such as gene copy number gains or losses and allele-specific imbalances [1]. Array-based comparative genomic hybridization (aCGH) [2] has, since its introduction, become a widely adopted tool for identification and quantification of DNA copy number alterations (CNAs) in tumor genomes [3]. The introduction of whole genome genotyping (WGG) arrays based on single nucleotide polymorphism (SNP) genotyping [4,5] allows for combined DNA copy number (SNP-CGH) and loss-of-heterozygosity (LOH) analysis at high resolution [6]. Current SNP arrays can genotype several hundreds of thousands of SNPs simultaneously. LOH analysis has in the past been a vital tool for the discovery of chromosomal regions harboring tumor-suppressor genes when inactivated by the classic mechanism of allelic loss [7]. LOH occurs as a consequence of reduction in copy number in a diploid genome but it may also appear as copy number-neutral LOH resulting from uniparental disomy or mitotic recombination events. The latter type of changes is not detectable by conventional aCGH platforms. Moreover, increases in copy number due to, for example, mono-allelic amplification may falsely be detected as LOH [8]. Therefore, by combining LOH and copy number analysis, regions of LOH derived from either copy number loss or neutral events may be identified. Conventional LOH studies compare the genotype of a tumor to its matched constitutional genotype. Current generations of WGG arrays have been reported to provide sufficiently high marker density to infer regions of LOH by the absence of heterozygous loci without the use of a matched control [9]. However, the increased marker density disqualifies the assumption of independence between allele calls of adjacent SNPs due to linkage disequilibrium. This may lead to detection of non-tumor specific homozygous regions based solely on the marker density. In the absence of a matched normal, haplotype correction methods may be required to remove such non-informative regions [9]. WGG arrays may eventually replace conventional aCGH platforms based on bacterial artificial chromosome clones or oligonucleotides due to their ability to generate both copy number and genotyping data [6]. However, this presumption has not been thoroughly investigated.

As previously described, allelic imbalances can conveniently be visualized in B allele frequency (BAF) plots representing the proportion of the two investigated alleles [6]. In BAF plots a value of 0.5 indicates a heterozygous genotype (AB), whereas 0 and 1 indicate homozygous genotypes (AA and BB, respectively). In a normal sample, three bands are expected in the BAF plot, a band centered at 0.5 for heterozygous SNPs, a band at 0 for SNPs genotyped as AA and a band at 1 for SNPs genotyped as BB. Allelic imbalances in tumor samples are observed in BAF plots as a deviation from 0.5 of SNPs heterozygous in cells with constitutional genotype. Detection of regions with LOH or allelic imbalance from WGG data has frequently been performed by methods incorporating hidden Markov models (HMMs) for which several different software packages exist, for example, dChipSNP [10], CNAT [11], PennCNV [12] and QuantiSNP [13]. Unfortunately, several of the existing software packages for LOH detection are currently only applicable for use with one of the two widely used WGG platforms, either Affymetrix or Illumina.

WGG arrays are increasingly employed for the analysis of tumor specimens. However, such samples often contain normal cell components and tumor cell subpopulations causing a dilution of tumor cell-specific imbalances. Such dilution reduces the sensitivity in LOH detection using SNP call-based methods [14]. Dilution of tumor cell specific allelic imbalances is seen in BAF plots as a compression of the split heterozygous populations towards the heterozygous center (at BAF = 0.5). Different methods have been proposed as solutions for Affymetrix GeneChip SNP arrays [14-16]. For Illumina, SOMATICs [17] was recently reported to allow for detection of allelic imbalance in tissues containing 40-75% tumor cells.

Here we describe a segmentation-based strategy for detection of LOH and allelic imbalances from WGG array data. The strategy allows for a large proportion of normal cell components and/or tumor cell clone heterogeneity. Transformation of B allele frequency profiles into a data representation free of allele association together with removal of non-tumor specific homozygous SNPs allows for direct application of segmentation algorithms from DNA copy number analysis, for example, circular binary segmentation (CBS) [18]. Segmented regions of similar allelic proportion are called as allelic imbalance by comparison to either a fixed threshold or a sample adaptive threshold as proposed for the normalization of copy number data [19]. Furthermore, the segmented value of an allelic imbalance can be used for accurate estimation of the proportion of affected cells.

We tested the performance of the segmentation strategy in simulated Illumina WGG data and in five experimental tumor WGG data sets. The results are compared to several other reported methods. The investigated data sets contain both paired tumor-normal samples, as well as unpaired tumor samples obtained from primary solid tumors and leukemias. The included tumors display a large set of different CNAs, including high level amplifications and homozygous deletions, as well as varying tumor heterogeneity and normal cell contamination. The data sets were generated on Illumina Genotyping BeadChips (300k, 370k and 550k) as well as on Affymetrix GeneChipArrays (250k), demonstrating the applicability of the segmentation strategy to different WGG platforms. Compared to currently used methods, we demonstrate that the proposed segmentation strategy has a high sensitivity and specificity for detecting allelic imbalances originating from DNA copy number gain, loss, and neutral events in heterogenic tumor specimens. We also demonstrate that the segmentation strategy can be used to accurately estimate the fraction of cells affected by allelic imbalance.

## Results and discussion

This study is outlined as follows with results and discussion presented accordingly. First we demonstrate that segmentation methods used in DNA copy number analysis can directly be applied to matched tumor-normal samples for identification of regions of similar allelic proportions. Next, the segmentation approach is generalized for use with unpaired tumor samples. The performance of the segmentation strategy in comparison to other methods is comprehensively evaluated using simulated as well as experimental data sets from different Illumina WGG platforms. Then, we describe how the segmentation approach with high accuracy and sensitivity detects and estimates the fraction of cells affected by an allelic imbalance. Finally, we describe how the segmentation approach can be adapted to Affymetrix WGG data.

### Segmentation identifies regions of identical allelic proportions in matched tumor-normal samples

**Figure 1:**
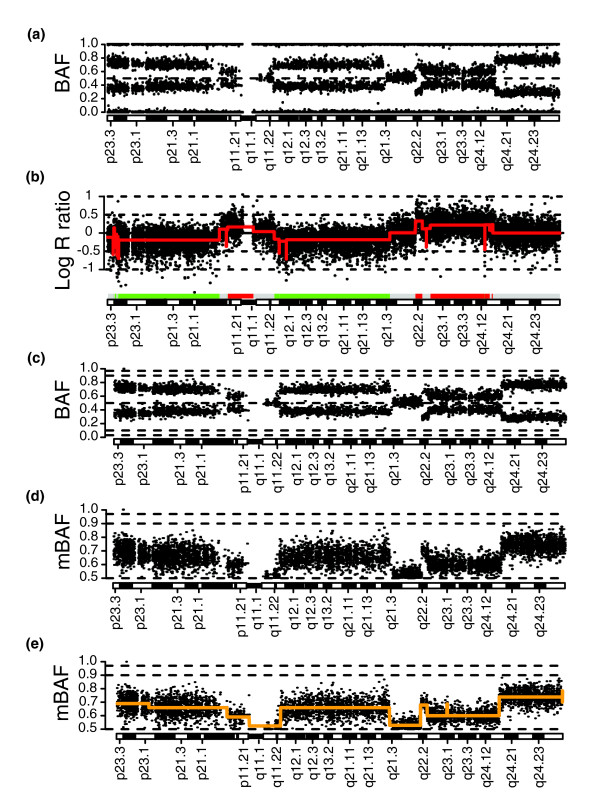
Transformation of B allele frequency data for a paired tumor sample. **(a) **BAF for chromosome 8 of breast tumor 2 (data set 1). **(b) **Copy number profile of chromosome 8 with CBS segmentation profile superimposed in red. Gains (red bars) and losses (green bars) are called by comparison of the CBS profile to log_2_-ratio thresholds (± 0.15). **(c) **B allele frequency for chromosome 8 with SNPs homozygous in the matched normal sample removed. Horizontal dashed lines indicate positions of 0.97, 0.9, 0.1, 0.03 and 0.5 in BAF. **(d) **Transformation of BAF into mBAF for chromosome 8. SNPs homozygous in the matched normal sample removed. Horizontal dashed lines indicate positions of 0.97, 0.9 and 0.5 in mBAF. **(e) **Segmentation of a paired breast cancer mBAF profile. CBS was applied to mBAF data for chromosome 8 of breast tumor 2 (data set 1) after removal of SNPs homozygous in the matched normal sample. CBS segmentation profile is superimposed in orange. Horizontal dashed lines indicate positions of 0.97, 0.9 and 0.5 in mBAF.

Allelic imbalances in tumor samples may conveniently be displayed using BAF plots, which illustrate the presence and location of genomic regions of apparently the same allelic proportion (Figure 1a). The nature of an allelic imbalance may be revealed by comparison to the corresponding copy number profile (Figure 1b). In conventional LOH analysis a matched normal sample is used for detection of LOH. SNPs that are homozygous in constitutional cells are non-informative for LOH analysis. For paired tumor-normal samples analyzed using WGG platforms, non-informative homozygous SNPs may be identified and removed by comparison of SNP genotype calls between the tumor and the matched normal, resulting in a tumor-specific BAF profile (Figure 1c). Furthermore, since alleles for SNPs are, with respect to haplotypes, arbitrarily called A or B, a set of genomically consecutive SNPs will appear in BAF plots as horizontal bands that are expected to be symmetrically positioned around 0.5. By performing a reflection of BAF data along the 0.5 axis, we obtain mirrored BAF (mBAF) estimates resembling a copy number profile (Figure 1d). Homozygous SNPs (AA or BB) are thus positioned at 1, while heterozygous SNPs are positioned at 0.5. A similar transformation was used in the recently reported SOMATICs algorithm [17].

In DNA copy number analysis, segmentation methods such as CBS [18] have been extensively tested for their ability to identify CNAs [20]. CBS can be directly applied to the mBAF tumor profile in Figure 1d to identify the breakpoints of the observed allelic imbalances (Figure 1e). When comparing the segmented mBAF profile (Figure 1e) to the copy number profile (Figure 1b) we find that the segmentation accurately detects regions of allelic imbalance due to copy number loss on 8p23.3 to 8p12 and 8q11.23 to 8q21.3, allelic imbalance due to copy number gain on 8p11.23 to 8p11.21 and 8q22.2 to 8q24.12, and apparent copy neutral LOH on 8q24.13 to 8q24.3. In conclusion, we find that a segmentation-based approach can be applied to Illumina WGG data to identify regions of allelic imbalance in matched tumor-normal samples.

### Generalization of the segmentation approach to unpaired tumor samples

The initial step in the segmentation approach is to remove non-informative homozygous SNPs from the tumor mBAF profile. Thus, generalization of the segmentation approach to unpaired tumor samples requires identification of non-informative SNPs when a matched normal sample is not available. Since the B allele frequency is a quantitative estimate of the allelic proportion for a given SNP, expected mBAF values for different types of allelic imbalances can be calculated for diploid genomes. An estimate of the tumor content of the analyzed sample can thus be translated into a maximal obtainable expected mBAF value for different types of allelic imbalances. The highest expected mBAF value, 1, is obtained for hemizygous loss or copy neutral LOH in a sample with 100% tumor content and no tumor heterogeneity. The highest achievable expected mBAF value decreases when contaminating normal cells and/or tumor cell sub-clones are present.

**Figure 2:**
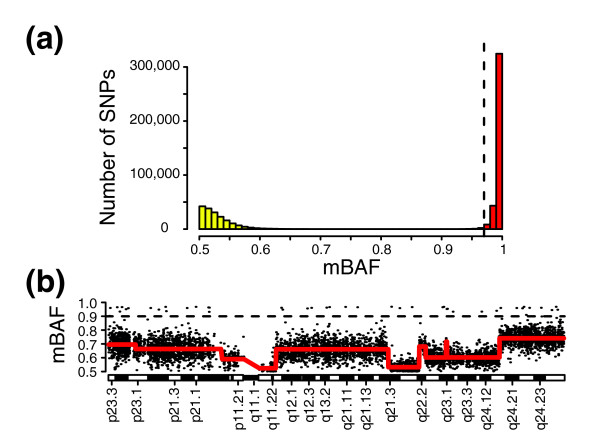
Generalization of the segmentation approach to unpaired tumor samples using a fixed mBAF threshold. **(a) **Histogram of mBAF values for the HapMap sample NA06991 (reference data set 4) hybridized on an Illumina Infinium 550k BeadChip. Bins with homozygous SNPs (AA and BB) are colored red. Bins containing heterozygous SNPs are colored yellow. **(b) **mBAF profile of chromosome 8 for breast tumor 2 (data set 1) with SNPs >0.97 in mBAF removed. CBS segmentation profile is superimposed in red. Horizontal dashed line indicates position of 0.9 in mBAF.

An estimation of tumor content can be used for generalization of the segmentation approach to unpaired tumor samples. Based on tumor content, the maximal obtainable expected mBAF value can be calculated and SNPs above this value can be removed as in the procedure for matched tumor-normal samples. For example, SNPs informative for a hemizygous deletion are, on average, not expected to obtain mBAF values larger than 0.91 for tumor samples with 10% normal cell contamination. On the other hand, for samples of purity above approximately 95%, using a fixed mBAF threshold for removal of non-informative homozygous SNPs may be inappropriate. The reason is that the range in mBAF of SNPs homozygous in all analyzed cells is often 0.97 to 1, as seen for normal samples analyzed on Illumina BeadChips (Table 1, Figure 2a). This variation makes non-informative homozygous SNPs difficult to distinguish from SNPs affected by tumor specific allelic imbalances for pure tumor samples. Still, for tumor samples of purity below 90-95%, or tumor samples of higher purity but with tumor cell subpopulations, a fixed mBAF threshold is an effective single parameter method for removing non-informative homozygous SNPs.

**Table 1:** mBAF statistics for homozygous SNPs in HapMap samples analyzed on Illumina BeadChips

Data set	Illumina platform	Number of samples	95^th ^percentile mBAF_AA+BB_	99^th ^percentile mBAF_AA+BB_	Mean mBAF_AA _± SD	Mean mBAF_BB _± SD
Reference 1	300k v1	111	0.99	0.973	0.998 ± 0.006	0.998 ± 0.006
Reference 2	300k v2	120	0.989	0.968	0.997 ± 0.005	0.998 ± 0.006
Reference 3	370k	123	0.98	0.961	0.993 ± 0.008	0.996 ± 0.009
Reference 4	550k	120	0.982	0.966	0.993 ± 0.008	0.998 ± 0.006

SD, standard deviation.

Applying a maximal mBAF cut-off of 0.97 to breast tumor 2 for removal of non-informative homozygous SNPs followed by segmentation results in a similar segmentation profile (Figure 2b) as when using the paired normal sample (Figure 1e). However, a fixed threshold may not fully remove non-informative SNPs if it is set too high. See, for example, Figure 2b, where some SNPs with high mBAF values (mBAF >0.9) are not removed compared to the matched case (Figure 1e). To remove such remaining non-informative SNPs, we first identify them by the absolute sum of the difference in mBAF between an investigated SNP and the SNPs that, in the maximal mBAF filtered data, precede and succeed the SNP. Next, SNPs having a deviation in mBAF from their neighboring SNPs larger than a set threshold are removed. This filtering process, herein referred to as triplet filtering (see Materials and methods), is illustrated in Figure S1 in Additional data file 1. To systematically evaluate the effect of triplet filtering, we applied it to the paired urothelial tumors in data set 2. We found that the addition of triplet filtering significantly improved the removal of non-informative SNPs (Figure S1 in Additional data file 1; Additional data file 2). In conclusion, the segmentation strategy can be generalized for unpaired tumor analysis by filtering out putative non-informative homozygous SNPs based on their mBAF values. Furthermore, normal cell contamination is advantageous for the segmentation strategy in unpaired tumor analysis, as the analyzed cells are a mix of cells with allelic imbalance (tumor cells) and cells with no imbalance (matched normal cells). This mix results in a compression of BAF estimates that distinguishes tumor-specific regions of allelic imbalance from non-informative regions of homozygosity.

### Calling of segmented regions as allelic imbalance

As illustrated in Figures 1e and 2b, segmentation can delineate regions of apparently the same allelic proportions for both paired and unpaired tumor samples. To differentiate regions of allelic imbalance from the heterozygous state, we can apply similar approaches as for calling CNAs from segmented data in DNA copy number analysis. In its simplest form we use a fixed mBAF threshold to compare segmented values against. If the segmented value of a genomic region is above the threshold, it is called as allelic imbalance. A fixed mBAF threshold may be given biological meaning through the equations giving expected mBAF values for different types of allelic imbalances (see Materials and methods). For example, to detect hemizygous loss in 20% of analyzed cells implies a maximum mBAF threshold of 0.56. We may also employ a sample adaptive approach for estimating the mBAF threshold as described for copy number analysis [19].

**Figure 3:**
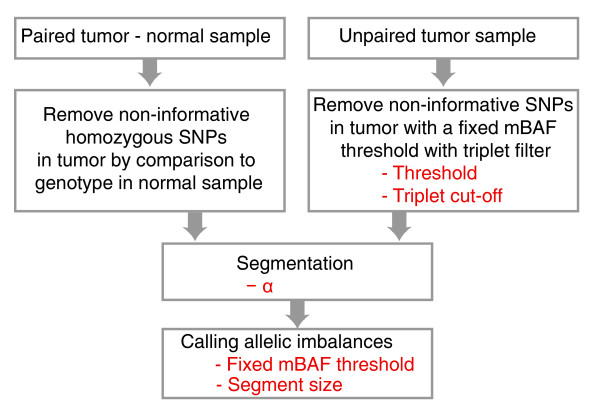
Flow chart of the analysis steps for the segmentation approach with parameters (in red) for paired and unpaired tumor analysis.

Figure 3 shows a schematic overview of the analysis steps in the segmentation approach with parameters for paired and unpaired tumor analysis. Using fixed thresholds, the number of parameters to optimize is typically one for paired tumor analysis (threshold for calling allelic imbalance) and two for unpaired analysis (threshold for removing non-informative SNPs and threshold for calling allelic imbalance). For the Illumina data sets we have analyzed, we have not found that other parameters (triplet-filtering cut-off, segmentation algorithm parameters, and minimum segment size) need to be tuned. If the threshold for removing non-informative SNPs in an unpaired analysis is set too high, a large number of non-informative SNPs may, for noisier samples, remain in the tumor mBAF profile. Such SNPs may form non-informative homozygous regions detected by the segmentation and falsely identified as regions of allelic imbalance. If the threshold is not optimized properly, haplotype correction [9] or size filtering of segments with high mBAF values needs to be employed to reduce the number of such false positive calls. When the tumor content of the analyzed cells is known, false positive segments can be filtered out on the basis of their segmented mBAF values.

### Evaluation and comparison of sensitivity and specificity using simulated Illumina data

To investigate the sensitivity and specificity of the segmentation approach compared to other methods, we created a simulated data set based on experimental 550k Illumina data for HapMap sample NA06991 (as described in Additional data file 3). Briefly, to the diploid HapMap sample we added a number of different CNAs and regions of copy neutral LOH to mimic a tumor sample. The simulated tumor sample was next diluted with normal cells creating a dilution series ranging from 0-100% tumor cell content in 5% increments. The ability to detect SNPs in allelic imbalance was evaluated for the segmentation strategy in both a paired and an unpaired setting. The performance of the segmentation strategy was compared with three published copy number variation (CNV) or allelic imbalance algorithms: PennCNV [12], QuantiSNP [13] and SOMATICs [17]. PennCNV and QuantiSNP are HMM-based methods developed for CNV analysis and should only detect allelic imbalances originating from DNA copy number gain and loss, whereas SOMATICSs also detects copy neutral allelic imbalances.

First, we evaluated whether the methods identified regions of allelic imbalance regardless of whether the methods also correctly identified the type of aberration (gain, loss or copy neutral). We calculated sensitivities for each allelic imbalance and overall specificities using SNPs heterozygous in the original HapMap sample. In this analysis, the sensitivity for a simulated allelic imbalance is the fraction of its SNPs that are called as allelic imbalance, and the overall specificity is the fraction of SNPs outside of all simulated allelic imbalances that are not called.

**Figure 4:**
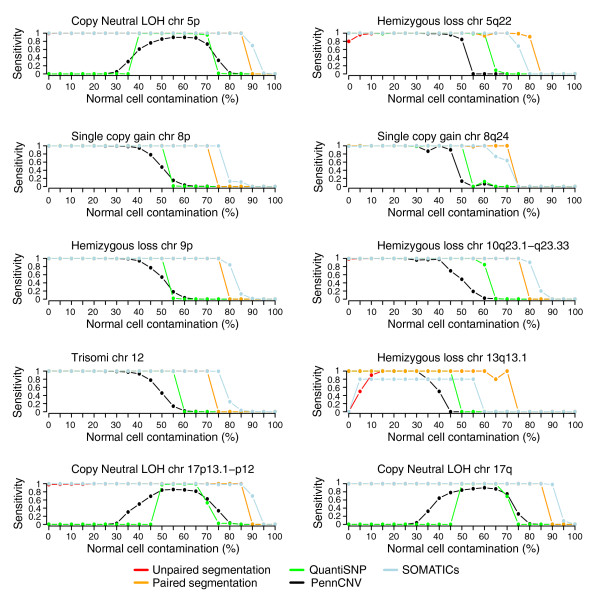
Comparison of sensitivity for detecting ten simulated allelic imbalances for different methods. Heterozygous SNPs in NA06991 were used to estimate the sensitivity for the methods in detecting allelic imbalances in the simulated data set with increasing normal cell contamination. Sensitivity was calculated for each method based on calls for allelic imbalance or not. Lines correspond to sensitivity for PennCNV (black), QuantiSNP (green), unpaired segmentation (red), paired segmentation (orange), and SOMATICs (blue).

**Figure 5:**
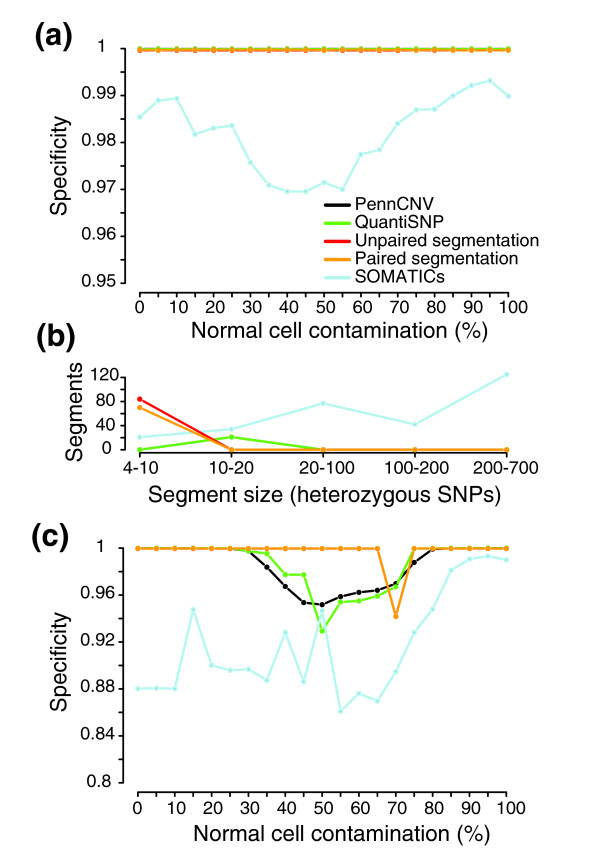
Comparison of specificity for detecting simulated allelic imbalances for different methods. Heterozygous SNPs in NA06991 were used to estimate the specificity of methods for detecting allelic imbalances with increasing normal cell contamination in the simulated data set. **(a) **Specificity for calls of allelic imbalance or not in the simulated data set. Lines correspond to specificity for PennCNV (black), QuantiSNP (green), unpaired segmentation (red), paired segmentation (orange), and SOMATICs (blue). **(b) **Size and number of erroneously called regions for PennCNV (black), QuantiSNP (green), unpaired segmentation (red), paired segmentation (orange), and SOMATICs (blue) across the entire simulated data set. Segment size is in consecutive erroneously called heterozygous SNPs. Only regions larger than four heterozygous SNPs are shown. **(c) **Specificity for correct calling of the type of allelic imbalance in the simulated data set. Lines correspond to specificity for PennCNV (black), QuantiSNP (green), unpaired segmentation (red), paired segmentation (orange), and SOMATICs (blue).

Sensitivities for detecting simulated allelic imbalances regardless of whether the correct type of aberration was identified are shown in Figure 4. For lower normal cell contaminations (<40%), all methods showed high sensitivity and concordance for detecting allelic imbalance originating from copy number gains and losses. For higher normal cell contaminations the segmentation strategy outperformed both PennCNV and QuantiSNP in both a paired and an unpaired analysis setting. Compared to SOMATICs, the segmentation strategy showed similar sensitivity throughout the dilution range. Even though PennCNV and QuantiSNP should not detect copy neutral events, we note that reducing calling to allelic imbalance or not cause both methods to erroneously detect copy neutral LOH regions, for example, chromosome 5p. The overall specificity was high (>99.99%) for PennCNV, QuantiSNP and the segmentation strategy across the dilution range (Figure 5a). SOMATICs showed the lowest specificity across the dilution range (ranging from approximately 97% to 99%), mainly due to a large number of erroneously called SNPs in the so-called red band of the algorithm. Additionally, SOMATICs identified the largest erroneously called segments, ranging up to larger than and exceeding 500 heterozygous SNPs in size (Figure 5b). Hence, SOMATICs obtains sensitivities similar to the segmentation strategy at the expense of identifying a larger number of false positive regions.

The detection of copy neutral imbalances using PennCNV and QuantiSNP led us to evaluate whether the methods, when they identify a region in allelic imbalance, also call the correct type of the aberration (gain, loss or copy neutral). In this second evaluation, the sensitivity for a simulated allelic imbalance is the fraction of its SNPs that are called as the correct type of imbalance. The overall specificity is calculated as in the previous evaluation with the addition that SNPs within an imbalance called as the incorrect type also contribute to lowering the overall specificity. For the segmentation strategy we used fixed cut-offs for the average log R ratio of SNPs in regions called as allelic imbalance to also call the type of aberration (see Materials and methods). The segmentation strategy had higher sensitivity than SOMATICs for correctly identifying gains and losses (Figure S2 in Additional data file 1). The CNV calling algorithm in SOMATICs repeatedly failed to call several regions of gain and loss correctly. Compared to only identifying allelic imbalance, the overall specificity for correct identification of the type of simulated allelic imbalance was considerably lower for PennCNV, QuantiSNP and SOMATICs, whereas it was high for the segmentation strategy also in this case (Figure 5c).

The segmentation strategy was, with the simulated data, able to detect regions of copy neutral LOH when the tumor content was only 15%. For hemizygous loss the maximum normal cell contamination that allowed detection was 75-80%, which corresponds well to the used mBAF threshold of 0.56 for calling allelic imbalance (hemizygous loss in >21% of analyzed cells). Single copy gain was detected with up to 75% normal cell contamination. Differences in sensitivity between paired and unpaired segmentation were seen for small allelic imbalances in samples of high tumor content. The low sensitivity for the 126 kb hemizygous loss on 13q13.1 for unpaired segmentation with 0-10% normal cell contamination is due to the fixed mBAF threshold of 0.97 for removing putatively non-informative homozygous SNPs (Figure 4). With this threshold value several of the tumor-specific homozygous SNPs for this CNA are removed, making it difficult to detect by segmentation.

BAF and copy number profiles for the simulated data set with regions called as allelic imbalance marked for PennCNV, QuantiSNP, SOMATICs, unpaired segmentation, and paired segmentation are available as described in Additional data file 4. In conclusion, we find that the segmentation strategy can sensitively detect different types of allelic imbalances in highly heterogeneous samples and perform well compared with other published methods.

### Evaluation and comparison of sensitivity using an experimental Illumina dilution series

**Figure 6:**
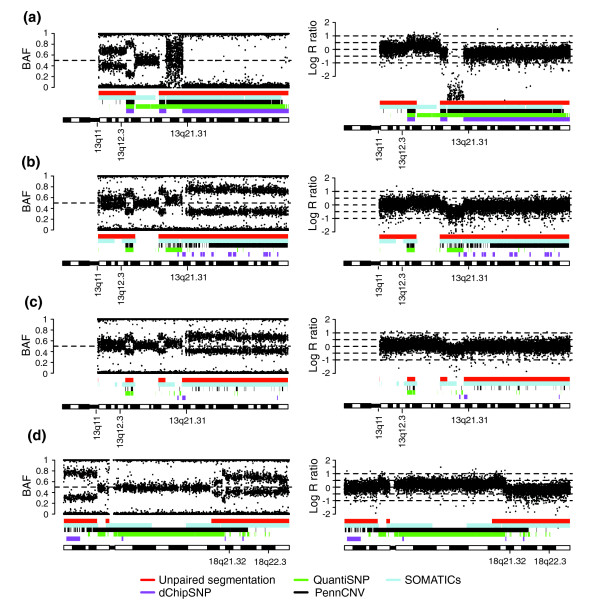
Allelic imbalances in CRL-2324 cells used for estimation of tumor dilution percentage by segmentation. CRL-2324 breast cancer cells were hybridized on Illumina 370k BeadChips in a dilution series with matched normal DNA (data set 3). For all parts, the left panel shows B allele frequency estimates and the right panel log R ratios. Bars indicate allelic imbalances detected by unpaired segmentation (red), SOMATICs (blue), PennCNV (black), QuantiSNP (green) and dChipSNP (purple). **(a) **Copy neutral LOH on 13q21.31-qter and single copy gain on 13q11-q12.3 in 100% CRL-2324 cells. **(b) **Copy neutral LOH on 13q21.31-qter and single copy gain on 13q11-q12.3 with 50% tumor fraction. **(c) **Copy neutral LOH on 13q21.31-qter and single copy gain on 13q11-q12.3 with 30% tumor fraction. **(d) **Hemizygous loss on chromosome 18q21.32-q22.3 with 50% tumor fraction.

To investigate the ability of the segmentation approach to detect allelic imbalances in experimental Illumina data, we generated a dilution series of the CRL-2324 breast cancer cell line on Illumina 370k BeadChips (data set 3). In addition to the methods applied to the simulated data (segmentation, PennCNV, QuantiSNP, and SOMATICs), we also included dChipSNP in this comparison. Since dChipSNP is a SNP genotype call-based method it could not be applied to the simulated data in which genotype calls were not simulated. CRL-2324 cells display a complex genetic make-up with polyploid cell populations having varying ploidy indices [21]. Aneuploidy may confound normalization and data interpretation of Illumina WGG data [6]. Normalization of Illumina WGG data in BeadStudio is made under the assumption that homozygous SNPs exist, on average, in two copies [6], an assumption that can lack validity for aneuploid tumor samples. Substantiating this concern, we observed for the CRL-2324 dilution series that BeadStudio normalization results in copy number profiles that are centered differently as the tumor content decreases (Figure 6a-c). As a consequence of this variation in centering, many of the methods will call the same type of allelic imbalance differently (gain, loss, or copy neutral) depending on how much the tumor is diluted. Therefore, we evaluated the methods using calls of allelic imbalance without regarding the type of aberrations.

**Figure 7:**
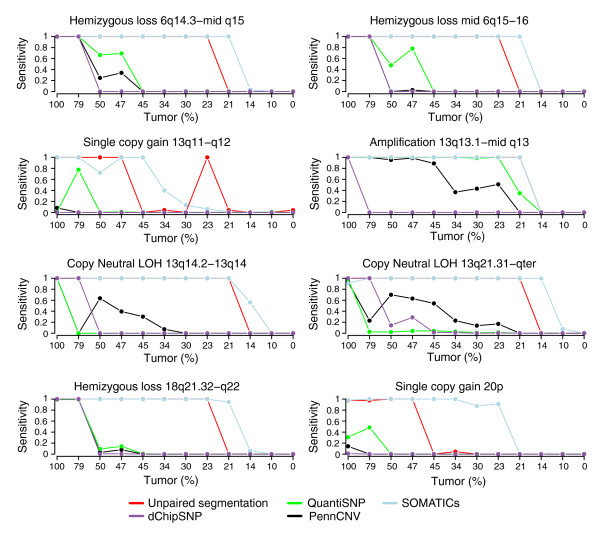
Comparison of sensitivity for detecting eight different allelic imbalances in the CRL-2324 dilution series for five methods. Lines correspond to sensitivity for PennCNV (black), QuantiSNP (green), unpaired segmentation (red), SOMATICs (blue), and dChipSNP (purple).

Sensitivity was determined for eight different CNAs having BAF values in the undiluted cancer cell line consistent with presence in all tumor cells (Figure 7). We found that the segmentation approach outperformed PennCNV, QuantiSNP and dChipSNP in sensitivity when tumor content was less than 50%. SNP call-based methods, such as dChipSNP, have been reported to be unable to detect regions of LOH when tumor content is less than 50% (corresponding to an mBAF of 0.66 for hemizygous loss), despite available paired constitutive DNA [14]. Aneuploidy is problematic for model-based HMM methods when detecting allelic imbalances. For example, using Penn CNV and QuantiSNP, the single copy gain on chromosome 13q11-q12.3 is not detected in the pure breast cancer cell line (Figures 6a and 7). This failure is a consequence of how BeadStudio centers the copy number profile. A further investigation of the normalization of tumor samples analyzed on Illumina WGG arrays is thus warranted. In concordance with the simulated data, the segmentation approach showed similar sensitivity as SOMATICs with decreasing tumor content for all allelic imbalances; except for the single copy gain on chromosome 20p, which was better detected by SOMATICs (Figure 7).

### Application of the segmentation approach to experimental Illumina tumor data sets

**Figure 8:**
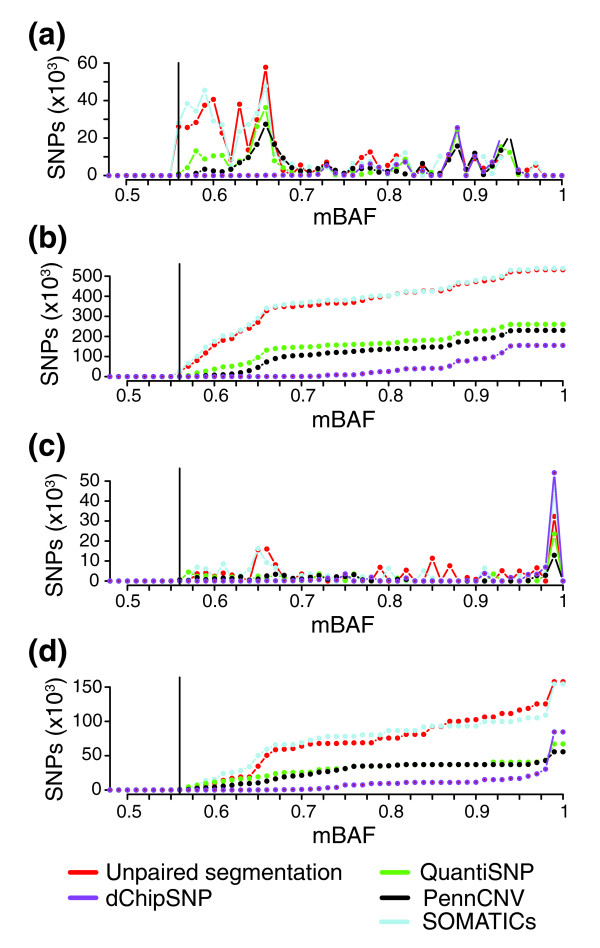
Total number of tumor specific SNPs detected as allelic imbalance in two paired tumor data sets plotted against their mBAF values for five methods. From each tumor, SNPs homozygous in the matched blood were removed. Only SNPs in segments of allelic imbalance >5 SNPs in size and with an average mBAF value ≥0.56 were counted and summarized across all samples in a data set. Lines correspond to the different methods: PennCNV (black), QuantiSNP (green), unpaired segmentation (red), SOMATICs (blue), and dChipSNP (purple). Vertical solid line corresponds to 0.56 in mBAF. **(a) **Total number of SNPs detected as allelic imbalance at different mBAF levels for the paired urothelial tumor data set (data set 2). **(b) **Cumulative number of SNPs detected at different mBAF levels for the paired urothelial tumor data set. **(c) **Total number of SNPs detected as allelic imbalance at different mBAF levels for the paired breast/colon tumor data set (data set 1). **(d) **Cumulative number of SNPs detected at different mBAF levels for the paired breast/colon tumor data set.

To investigate the performance of the segmentation approach in solid tumors, we applied it to two data sets containing matched tumor-normal samples (data sets 1 and 2). By removal of SNPs homozygous in the paired normal sample we generated a tumor specific BAF profile for each sample (as in Figure 1c), which was transformed to an mBAF profile (as in Figure 1d). A method for sensitive detection of allelic imbalances in tumors should detect genomic regions containing SNPs with small but distinct differences in mBAF compared to the 0.5 mBAF baseline. Consequently, to compare methods, we calculated the number of SNPs detected as allelic imbalance across a data set for different tumor specific mBAF values (Figure 8). We found that the segmentation strategy outperforms PennCNV, QuantiSNP and dChipSNP for both data sets in detecting SNPs at lower mBAF values. The segmentation strategy performs similar to SOMATICs in both data sets down to mBAF values as low as 0.56, which was used as the cut-off to call allelic imbalance in the segmentation strategy. Paired BAF and copy number profiles for seven paired tumor samples (data sets 1 and 2) with regions called as allelic imbalance marked for PennCNV, QuantiSNP, dChipSNP, SOMATICs, and unpaired segmentation are available as described in Additional data file 4.

**Figure 9:**
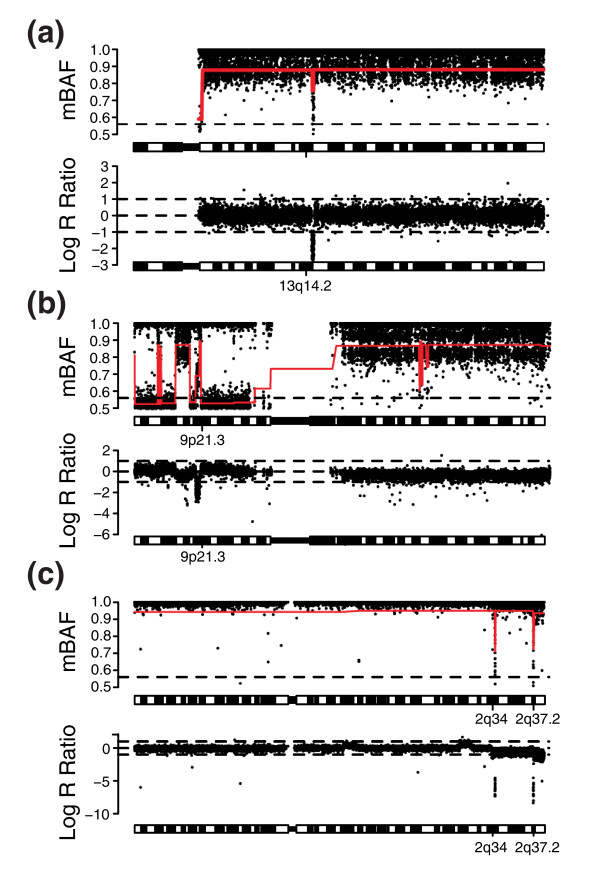
Detection of homozygous deletions in various tumor samples by the segmentation approach. All samples are hybridized on Illumina 300k or 370k BeadChips. For all parts, the upper panel shows the mirrored B allele frequency profile and the bottom panel shows the copy number profile. Red lines represents the CBS segmentation profile. Horizontal dashed lines in the mBAF panel represents the threshold for calling allelic imbalance (0.56). **(a) **Chromosome 13 of CLL sample 7 (data set 4) with a homozygous deletion on 13q14 in 80% of analyzed tumor cells. **(b) **Homozygous deletion of CDKN2A on chromosome 9p21.3 in urothelial tumor UC211_R (data set 2). **(c) **Homozygous deletions on chromosome 2q34 and 2q37.2 in breast tumor 1 (data set 1).

Detection of homozygous deletions using the B allele frequency alone can be challenging [22]. In the case of complete homozygous deletion in all investigated cells no genetic material remains and the BAF estimates become essentially random due to the low SNP signal intensity [22]. With an increasing fraction of normal cell contamination, BAF estimates for homozygously deleted regions will eventually become indistinguishable from regions of 2N (Figure 6a-c). However, homozygous deletions frequently occur within regions of somatic LOH in tumor specimens. Such events can create a clearly distinguishable pattern detectable by the segmentation approach (Figure 9). Nevertheless, homozygous deletions are, in general, probably best detected from analyzing copy number ratios [6].

While the segmentation strategy is designed to identify LOH and allelic imbalances in heterogeneous cancer samples, germline CNVs can be either missed or detected depending on their genotype and size. Germline CNVs involving loss result in BAF profiles identical to hemizygous loss in pure tumor samples and hence may be detected due to the absence of heterozygous loci if the CNVs are sufficiently large. Small germline CNVs involving gain of genetic material are not detected if the affected SNPs only show a homozygous genotype (for example, AAA or BBB, giving mBAF values close to 1). Larger germline CNVs involving gain may be detected similarly as for tumors with gain of genetic material.

### Estimating cellular composition of samples from segmented B allele frequencies

BAF values in combination with copy number status allow for a direct estimation of the proportion of cells displaying a certain allelic imbalance [22]. For a diploid genome, theoretical BAF values for allelic imbalances such as single copy gain, hemizygous loss or copy neutral LOH can be determined for varying percentages of normal cell contamination. Furthermore, knowledge of the sample purity can be used to estimate the fraction of tumor cells affected by an allelic imbalance.

Two studies have used different approaches to demonstrate how BAF data can be used to estimate normal cell contamination for tumor samples [17,22]. We have derived equations for how mBAF values expected for different types of allelic imbalances depend on the fraction of cells harboring the imbalance (see Materials and methods). Nancarrow *et al. *[22] do not present equations, but for the different allelic imbalances in the simulated dilution series we obtain, using their software SiDCoN, theoretical BAF values identical to those obtained with our equations. We conclude that the equations we have derived are identical to the approach used by Nancarrow *et al*. Since the segmented mBAF value of a genomic region represents an average of the investigated SNPs, it can directly be used for estimation of the fraction of cells not affected by an allelic imbalance. We first evaluated the accuracy of the segmented value as a tool for estimation of tumor content in heterogeneous samples using the simulated data set. The simulated tumor content was compared to the value calculated from the observed segmented mBAF values for three different types of allelic imbalances. The segmentation approach finds the theoretical values with high accuracy and provides close estimates of the simulated tumor content (Table 2). The discrepancy for the unpaired tumor setting when tumor content is above 95% is due to the fixed mBAF threshold of 0.97 used to filter our SNPs believed to be non-tumor specific.

**Table 2:** Estimation of tumor cell content from simulated data using segmentation

Tumor	Tumor cells using hemizygous loss (%)*		Tumor cells using single copy gain (%)^†^		Tumor cells using copy neutral LOH (%)^‡^	
			
cells (%)	Paired	Unpaired	Paired	Unpaired	Paired	Unpaired
0	-	-	-	-	-	-
5	-	-	-	-	-	-
10	-	-	-	-	-	-
15	-	-	-	-	15	15
20	-	-	-	-	21	21
25	26	26	-	-	25	25
30	30	30	31	31	30	30
35	35	35	36	36	35	35
40	40	40	41	41	40	40
45	45	45	45	45	45	45
50	50	50	50	50	49	49
55	55	55	55	55	55	55
60	59	59	60	60	60	61
65	65	65	65	65	64	65
70	70	70	70	70	69	70
75	75	75	75	75	75	75
80	80	80	80	80	80	80
85	85	85	85	85	85	84
90	90	90	90	90	89	86
95	95	93	96	96	94	88
100	99	95	101	101	97	89

*Hemizygous loss on chromosome 10q23.1-q23.33. ^†^Mono allelic gain on chromosome 8p. ^‡^Copy neutral LOH on 17p13.1-p12. Dashes indicate 'not detected'.

**Figure 10:**
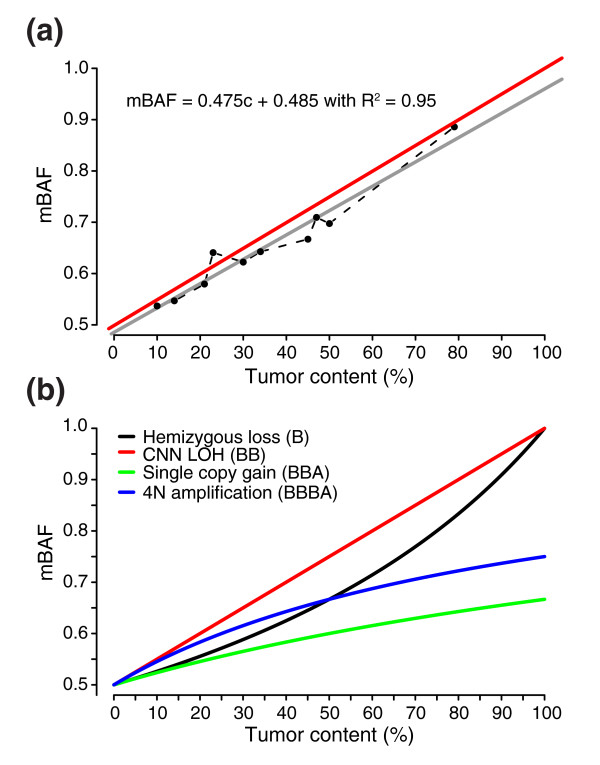
Theoretical and observed mBAF values for different types of allelic imbalances with increasing tumor content for the CRL-2324 dilution series. **(a) **Linearity of segmented mBAF values for the copy neutral LOH region on 13q21.31-qter across the CRL-2324 dilution series. The red line corresponds to expected mBAF values and the gray line to a linear regression fit for experimental data between 0% and 100% tumor content, for which c represents the tumor fraction. **(b) **Theoretical mBAF values for different allelic imbalances: hemizygous loss (black line), copy number neutral LOH (red line), single copy gain (green line), and 4N amplification, for example, BBBA (blue line).

To verify the accuracy of the segmented value in experimental Illumina data, we applied it to the CRL-2324 dilution series (data set 3). Three different allelic imbalances with 100% penetrance in CRL-2324 cells were selected (Figure 6) for comparing the tumor content estimated by segmentation with the dilution percentage. In concordance with the simulated data, we found that the segmentation approach provides close estimates of the theoretical mBAF values and can accurately estimate tumor content in experimental Illumina data (Table 3). The discrepancy for 100% tumor content is due to the fixed mBAF threshold of 0.97. Furthermore, the expected value for 0% tumor content is not in reality 0.5 due to the transformation from BAF to mBAF. The experimental CRL-2324 dilution series shows the expected linear compression of mBAF for the 13q21.31-qter copy neutral LOH region (Figure 10a). Tumor content appears to be best estimated from regions of hemizygous loss or copy neutral LOH, due to their larger span in mBAF (Figure 10b). Discrepancies between the dilution percentage and the estimated percentage from segmentation may in part be explained by uncertainty in the measured DNA content, which introduces bias in the expected dilution percentages (Table 3). Such bias may explain differences seen in sensitivity between the simulated data set (Figure 4) and the CRL-2324 dilution series (Figure 7) for low tumor contents. Due to the chosen mBAF threshold of 0.56 for calling allelic imbalance, hemizygous loss cannot be detected below 20%, and single copy gain not below 25% tumor content.

**Table 3:** Estimation of tumor content by segmentation in the CRL-2324 dilution series (data set 3)

	Hemizygous loss 18q21.32-q22.3^†^			Single copy gain 13q11-q12.3^‡^			CNN LOH 13q21.31-qter^§^		
			
Tumor (%)*	Expected mBAF	Observed mBAF	Tumor (%)	Expected mBAF	Observed mBAF	Tumor (%)	Expected mBAF	Observed mBAF	Tumor (%)
0	0.5	0.53	12	0.5	0.53	15	0.5	0.53	6
10 (7-12)	0.53	0.53	11	0.52	0.53	12	0.55	0.54	7
14 (10-17)	0.54	0.53	12	0.53	0.53	12	0.57	0.55	9
21 (17-26)	0.56	0.55	18	0.55	0.54	16	0.6	0.58	16
23 (18-28)	0.56	0.6	32	0.55	0.56	28	0.62	0.64	28
30 (24-35)	0.59	0.58	27	0.57	0.55	21	0.65	0.62	24
34 (28-40)	0.60	0.59	31	0.57	0.55	23	0.67	0.64	29
45 (38-51)	0.65	0.61	37	0.59	0.56	27	0.72	0.67	33
47 (40-54)	0.65	0.65	46	0.6	0.57	35	0.74	0.71	42
50 (43-57)	0.67	0.64	43	0.6	0.57	30	0.75	0.7	39
79 (74-83)	0.83	0.84	81	0.64	0.6	51	0.9	0.89	77
100	1	0.93	93	0.67	0.65	88	1	0.95	89

*95% confidence interval within parentheses. ^†^Hemizygous loss chr18:54400001-71300000. ^‡^Single copy gain chr13:16000001-31100000. ^§^Copy number neutral LOH chr13:60500001-114142980.

When the tumor content of the analyzed cells is known, the segmentation strategy can be used to estimate the tumor sub-clone content for allelic imbalances. A reported comparison of four different array platforms for detection of CNAs and LOH in chronic lymphocytic leukemias (CLLs) included fluorescent *in situ *hybridization (FISH) verifications of a number of hemizygous losses observed in tumor cell subpopulations [23]. We applied the segmentation strategy to the Illumina data from this CLL study (data set 4). Our results demonstrate that the tumor cell sub-clone content for hemizygous losses can be accurately estimated from the segmented mBAF value (Table 4). Furthermore, the percentage of cells affected by copy number neutral LOH can also be estimated using the segmented value. CLL sample 7 was shown to be copy neutral for chromosome 13, besides a homozygous loss of 13q14 [23] (Figure 9a). Of the tumor cells, 11% were found to have hemizygous loss of 13q14 and 80% to have homozygous loss by FISH [23]. However, the mBAF profile reveals allelic imbalance of the whole chromosome, implying copy neutral LOH (Figure 9a). Using the segmented value for chromosome 13, excluding 13q14, we estimated the percentage of tumor cells affected by the copy neutral LOH to be 83%. Intriguingly, this estimated percentage closely matches the fraction of tumor cells shown to have the homozygous loss by FISH (80%) [23]. This observation suggests that a small fraction of tumor cells carry only the hemizygous loss of 13q14 found by FISH, while the larger population has both the bi-allelic loss on 13q14 and loss of one allele followed by duplication of the remaining allele for chromosome 13.

**Table 4:** Estimation of tumor cell sub-clone content by segmentation for hemizygous loss in CLL samples

Sample*	CLL cells (%)^†^	Genomic region	Hemizygous loss by FISH (%)	Hemizygous loss by segmentation (%)^‡^
8	87	17p13.3-p11.1	73	77
9	90	11q22.1-q23.3	56	58
10	86	17p13.3-p11.2	90	94

*Data set 4. mBAF cut-off for each sample based on the maximum theoretical value for copy neutral LOH for the respective tumor content. ^†^Percent CLL cells (CD5^+^/CD23^+^) were estimated by flow cytometry. ^‡^The average segmented value for the region was used.

Estimation of tumor content is difficult and usually rare for solid tumors. Tumor content and tumor cell sub-clone content can be estimated with the segmentation approach under certain assumptions. For example, by assuming that a certain allelic imbalance occurs in all tumor cells, normal cell contamination becomes the sole driving force behind BAF compression. In this case, the tumor cell content can be estimated from the segmented value of the imbalance. Once the tumor cell content is estimated, the fraction of tumor cells affected by every other allelic imbalance can be calculated. In conclusion, we have shown that the segmentation strategy can be used to accurately estimate normal cell contamination and the fraction of cells affected by an allelic imbalance.

### Application of the segmentation approach to Affymetrix WGG data

Allelic imbalances for Affymetrix data are usually not displayed using BAF plots. BAF estimates can, however, be generated for Affymetrix WGG data in a similar fashion as for Illumina [23]. Technical variation in BAF estimates appears to differ between Affymetrix and Illumina WGG data as observed in Gunnarsson *et al*. [23]. The difference is further illustrated in Figure S3 in Additional data file 1 for an urothelial carcinoma hybridized on an Illumina 370k BeadChip and on an Affymetrix 250k Nsp array. The values for the thresholds in the segmentation strategy need to be modified in order for the strategy to handle the larger variation in Affymetrix BAF estimates (Figure S4 in Additional data file 1). Due to larger variation for homozygous SNPs, both the mBAF threshold and the triplet cut-off need to be reduced to filter out non-informative SNPs. As a consequence, the sensitivity is reduced for tumor samples of high purity. Additionally, the increased mBAF variation results in increased average values for segments. By replacing the mean with the median in the CBS algorithm when determining the segmented value for a genomic region, such increases can be counteracted.

**Figure 11:**
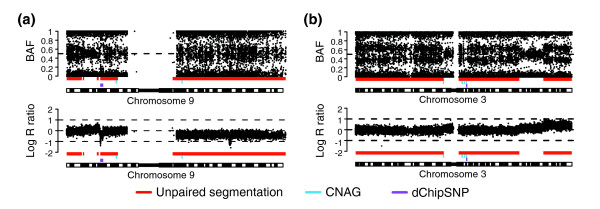
Application of the segmentation strategy to urothelial tumors hybridized on Affymetrix 250k Nsp arrays. Bars indicate allelic imbalances detected by unpaired segmentation (red), CNAG (blue), and dChipSNP (purple). In both parts, the top panel shows BAF estimates and the lower panel copy number estimates for two samples in data set 2. **(a) **Chromosome 9 of urothelial tumor UC1. **(b) **Chromosome 3 of urothelial tumor UC3.

Applying the segmentation strategy to two urothelial tumors analyzed on Affymetrix 250k Nsp arrays demonstrates how regions of allelic imbalance in solid tumors, missed by both dChipSNP and CNAG [24], can be detected (Figure 11). To estimate the tumor fraction affected by specific allelic imbalances, we applied the segmentation approach to Affymetrix data for CLL cases 8, 9 and 10 (data set 4) and investigated the same hemizygous deletions as we did using the Illumina data. For the hemizygous losses we obtained the tumor content estimates 75%, 56% and 85%, respectively, which are comparable to the results for the Illumina data (Table 4) and also closely match the FISH results. The percentage of tumor cells affected by copy neutral LOH on chromosome 13 in CLL sample 7 was, as for the Illumina data, estimated to be 83% using the segmented mBAF value. B allele frequency and copy number profiles for the two urothelial tumors in data set 5 with regions called as allelic imbalance marked for CNAG, dChipSNP, and unpaired segmentation are available as described in Additional data file 4. In conclusion, the segmentation approach can be applied to Affymetrix WGG data with modified parameter values to address the larger variation in BAF estimates for this platform.

## Conclusion

We demonstrate that a segmentation-based strategy may successfully be applied to WGG data for sensitive detection of regions affected by LOH or allelic imbalance in samples with a high degree of heterogeneity. The strategy can be applied to data derived from different WGG platforms both for unpaired and paired LOH analysis. We obtain results highly concordant with several other methods but with increased sensitivity and high specificity for detecting allelic imbalances in heterogeneous samples. We also demonstrate that the segmentation strategy can be used to identify allelic imbalances only present in sub-clones and to provide accurate estimates of the fraction of cells affected by allelic imbalances. The proposed segmentation strategy represents a valuable new platform independent tool for analysis of high density WGG data.

## Materials and methods

### Experimental data sets

We used five tumor data sets to evaluate and compare the proposed segmentation strategy together with reference data sets for the different Illumina platforms. Data set 1 consists of six hybridizations on Illumina HumanHap300 version 1 Genotyping BeadChips representing one colon cancer and two breast cancer tumors, with matched normal samples (Courtesy of Illumina Inc., San Diego, CA, USA). Data set 2 consists of 15 urothelial carcinomas hybridized on HumanCNV370 Genotyping BeadChips together with matched normal samples. Data set 3 consists of a dilution series for the breast cancer cell line CRL-2324 [21] hybridized on HumanCNV370 Genotyping BeadChips. Genomic DNA from CRL-2324 and its matched normal cell line (CRL-2325) was obtained from ATCC [25]. Dilutions (0, 10, 14, 21, 23, 30, 34, 45, 47, 50, 79 and 100% tumor DNA content) were made by mixing tumor DNA with normal matched DNA. DNA concentrations were determined using the Qubit picogreen fluorometric assay (Invitrogen, Carlsbad, CA, USA). To obtain confidence intervals for the tumor DNA content of the dilutions, a series of replicate measurements of DNA concentrations were performed and a coefficient of variation (CV) of 10% was obtained. This CV is similar to findings by others for picogreen assays [26,27]. A CV of 10% was, using error propagation, turned into an estimated standard deviation of the tumor DNA fraction for each dilution experiment. These standard deviations were turned into 95% confidence intervals using a normal approximation. Data set 4 consists of ten CLL cases hybridized on Illumina HumanHap300 version 2 Genotyping BeadChips and Affymetrix 250k Nsp arrays [23]. Data set 5 consists of two urothelial carcinomas obtained from the same patient and a matched normal sample hybridized on Affymetrix 250k Nsp arrays. Call rates for data set 5 were 97.2%, 97.3% and 95.9%, respectively, using the DM algorithm. Reference data set 1 consists of 111 HapMap [28] samples hybridized on Illumina HumanHap300 version 1 Genotyping BeadChips (Courtesy of Illumina Inc.). Reference data set 2 consists of 120 HapMap samples hybridized on Illumina HumanHap300 version 2 Genotyping BeadChips (Courtesy of Illumina Inc.). Reference data set 3 consists of 123 HapMap samples hybridized on Illumina HumanCNV370 Genotyping BeadChips (Courtesy of Illumina Inc.). Reference data set 4 consists of 120 HapMap samples hybridized on Illumina HumanHap550 Genotyping BeadChips (Courtesy of Illumina Inc.). Illumina hybridizations for data set 4 were performed at the SNP technology platform in Uppsala, Sweden [29] according to the manufacturer's instructions. Illumina hybridizations for data sets 2 and 3 and Affymetrix hybridizations for data set 5 were performed at the SCIBLU Genomics Centre at Lund University, Sweden [30] according to the manufacturer's instructions.

### Data preprocessing

For Illumina data, fluorescent signals were imported into the BeadStudio software (Illumina Inc.) and normalized. The normalized fluorescence signals for a sample were compared with the signal intensities of a set of reference genotypes, and the log_2_-ratios between the sample and the reference signals were calculated. In addition, the frequency of the B-allele for the sample was estimated based on the reference genotype clusters [6]. For Affymetrix data, quality control, genotype calling, and copy number analyses were made in the Affymetrix GeneChip^® ^Genotyping Analysis Software (GTYPE) 4.1. Genotype calls were made using the BRLMM algorithm [31]. The HMM algorithm in the Copy Number Analysis Tool (CNAT) 4.0.1 was used with the following parameter settings: transition decay 5 Mb, median normalization, and no smoothing to generate log_2_-ratio estimates for Affymetrix data. The reference data set for copy number analyses was 96 CEU samples from the HapMap project [28]. B allele frequencies for Affymetrix data were estimated as described [23].

BAF data were reflected into mBAF along the 0.5 axes by the transformation mBAF = abs(BAF - 0.5) + 0.5, where abs stands for taking the absolute value. Data for chromosomes 1-22 were used in subsequent comparisons.

### Equations for allelic imbalances in diploid genomes

Since BAF is a measurement of N_B_/(N_B _+ N_A_), where N_A _and N_B _are the number of alleles, a region of hemizygous loss can, for a diploid genome, be estimated to have an expected mBAF of 1/(1 + x), where x is the fraction of cells not showing the allelic imbalance, for example, contaminating normal cells. Similarly, a copy neutral event can be estimated to have an expected mBAF of (2 - x)/2 and a single copy gain to have an expected mBAF of (2 - x)/(3 - x). More complex aberrations, such as AAAB/BBBA, corresponding to 4N, can be estimated to have an expected mBAF of (3 - 2x)/(4 - 2x), AAABB/BBBAA to have (3 - 2x)/(5 - 3x), AAAAB/BBBBA to have (4 - 3x)/(5 - 3x), AAA/BBB to have (3 - 2x)/(3 - x), AAAA/BBBB to have (4 - 3x)/(4 - 2x), and AAAAA/BBBBB to have (5 - 4x)/(5 - 3x).

### Simulated data sets for evaluation of sensitivity and specificity

A simulated Illumina data set was created for evaluation of the sensitivity and specificity of the proposed segmentation method compared to PennCNV, QuantiSNP and SOMATICs. The simulated data set was based on the diploid HapMap sample NA06991 hybridized on an Illumina HumanHap550 Genotyping BeadChip (reference data set 4). Different types of allelic imbalances were added to NA06991 at distinct genomic locations using the equations for the theoretical mBAF levels of single copy gain, hemizygous loss and copy neutral LOH. The simulated data set consists of 21 versions of the modified NA06991 sample with varying degrees of normal cell contamination, starting from 0% up to 100% with 5% increments. The construction and analysis of the simulated data set is described in detail in Additional data file 3.

### DChipSNP, PennCNV, QuantiSNP, SOMATICs and CNAG analyses

Tumor-only LOH analysis was performed using the software dChipSNP [10] with the consider haplotype option for both Affymetrix and Illumina data. For Affymetrix, the reference data were 250k Nsp data from 60 CEPH parents [32]. For Illumina, the reference data were the 32 CEU parents selected from the HumanHap300 genotyping data set. Regions where LOH was called in more than 10% of the reference data were removed from further analysis. PennCNV analysis was performed with default settings (Additional data file 3) as previously described [12]. Only regions of copy number gain and loss are detected. QuantiSNP analysis (QuantiSNP ver 1.0) was performed with default settings (Additional data file 3) as previously described [13]. Only regions of copy number gain and loss are detected. GC correction was not employed. Calls were set as -1 (undefined), 2 (normal), 1 (copy number loss) or 3 (copy number gain). SOMATICs analysis was performed with default settings as previously described [17], with the exception that the average heterozygosity rate was set to 0.31 and that a BAF *p*-value of 0.05 was used to filter detected allelic imbalances. Modules 1 to 4 in the R script (Additional data file 3) from [33] were used for analysis. Calls were set as -1 (copy number loss), 0 (copy neutral allelic imbalance) or 1 (copy number gain). CNAG analysis was performed on Affymetrix data using version 2 of CNAG in an unpaired test setting [24]. For urothelial data set 5 the matched blood was set as an unpaired reference sample. Cut-off for the LOH likelihood was decreased to 5 for increased sensitivity compared to the default 30. For all other parameters default settings were used.

### SNP enrichment for segmentation of paired tumor samples

Non-informative homozygous SNPs were removed from matched tumor-normal samples by comparison of genotype calls. SNPs genotyped as AA or BB in the matched normal samples were removed from the corresponding tumor BAF profile before transformation to mBAF.

### SNP enrichment for segmentation of unpaired tumor samples

For unpaired tumor analysis non-informative homozygous SNPs may be removed from the tumor mBAF profile by using an mBAF threshold. SNPs above the threshold are considered non-informative and removed. Triplet filtering is next applied to the mBAF threshold filtered data to further improve the removal (Figure S1 in Additional data file 1; Additional data file 2). For each SNP the absolute sum of the difference in mBAF between the investigated SNP and the pre- and succeeding SNP (neighboring SNPs are identified in the mBAF threshold filtered data) is calculated and added to the SNPs distance from the 0.5 baseline. For a SNP with index i:

triplet sum[i] = abs(mBAF[i - 1] - mBAF[i]) + abs(mBAF[i + 1] - mBAF[i]) + mBAF[i] - 0.5

Triplet sums are compared against a threshold. SNPs with triplet sums above the threshold are considered outliers and removed. The triplet filtering is designed to remove non-informative homozygous SNPs that, due to experimental noise, obtain mBAF values lower than the mBAF threshold. The small numbers of remaining non-informative SNPs that are not removed by triplet filtering (Additional data file 2) include consecutive non-informative homozygous SNPs that all obtain mBAF values below the mBAF threshold. In this study we used an mBAF threshold of 0.97 and a triplet sum threshold of 0.8 for Illumina data. For Affymetrix data, we used an mBAF threshold of 0.9 and a triplet sum threshold of 0.6.

### Segmentation of allelic proportions

CBS [18] (DNAcopy [34]) was used to identify breakpoints of genomic regions of apparently identical allelic proportion. Segmentation was performed on mBAF profiles after removal of non-informative homozygous SNPs. In this study default settings of CBS were used, except for the significance level for accepting change-points (α), which was set to 0.001.

### Calling of allelic imbalances for the segmentation approach

Regions of allelic imbalance may be called by comparison of the respective segmented mBAF value to an mBAF threshold. Values above the threshold imply allelic imbalance. Thresholds may be either fixed or sample adaptive. Sample adaptive thresholds may be generated using enriched mBAF data similarly to as previously described for copy number analysis [19]. The AsCNAR software has been reported to be able to detect allelic imbalance with up to 80% contaminating normal cells for Affymetrix WGG arrays [14]. For hemizygous loss, this level of normal cell contamination corresponds to a theoretical mBAF value of 0.555. Consequently, in this study we used a fixed threshold in mBAF of 0.56 for both Affymetrix and Illumina data to call allelic imbalance. Segments called as allelic imbalance and smaller than four SNPs in size were removed from further analysis. A simple method for also calling the type of aberration (gain, loss, or copy neutral) for regions called as allelic imbalance by the segmentation strategy was constructed. Types of aberrations were called using fixed cut-offs for the average log R ratio of SNPs in regions called as allelic imbalance. The fixed mBAF threshold of 0.56 used to call allelic imbalance corresponds to a fraction of less than 80% unaffected cells for hemizygous loss. Similarly, this mBAF threshold corresponds to a fraction of 73% unaffected cells for single copy gain. These fractions can, utilizing an investigation of how log R ratios depend on copy numbers [6], be turned into the log R ratio cutoffs -0.15 and 0.073 for losses and gains, respectively (Additional data file 3). Consequently, we called allelic imbalances with an average log R ratio above 0.06 relative to the median log R ratio of the entire sample as gain, below -0.14 as loss, and in between as copy neutral.

### Availability

An implementation of the proposed segmentation strategy, BAFsegmentation, is available together with the simulated data set [35]. The implementation of the segmentation strategy can generate analysis bookmarks of identified regions for use with the Illumina BeadStudio software. Four matched tumor-normal pairs in data set 2 and the CRL-2324 dilution series (data set 3) are available through Gene Expression Omnibus [36] with accession GSE11976.

## Abbreviations

aCGH, array-based CGH; BAF, B allele frequency; CBS, circular binary segmentation; CGH, comparative genomic hybridization; CLL, chronic lymphocytic leukemia; CNA, copy number aberration; CNV, copy number variation; CV, coefficient of variation; FISH, fluorescent *in situ *hybridization; HMM, hidden Markov model; LOH, loss of heterozygosity; mBAF, mirrored B allele frequency; SNP, single nucleotide polymorphism; WGG, whole genome genotyping.

## Authors' contributions

JS conceived the study. JS developed and implemented the method and performed data analysis with assistance from MR. JS generated the experimental dilution series. JS, MR, JVC and DL proposed analyses and interpreted results. JS and MR wrote the manuscript. AI and HG performed dChipSNP analysis and calculated B allele frequencies for Affymetrix data. GJ, RR, MH and ÅB contributed samples. All authors approved the final manuscript.

## Additional data files

The following additional data are available. Additional data file 1 is a set of figures describing the implementation of the segmentation approach and analysis using the approach. Additional data file 2 is a table describing results using triplet filtering. Additional data file 3 is a document describing the generation and analysis of the simulated data set. Additional data file 4 is a document describing the analysis of experimental data available at the project web site [35].

## Supplementary Material

Additional data file 1 Implementation of the segmentation approach and analysis using the approach. Click here for file

Additional data file 2 Results using triplet filtering. Click here for file

Additional data file 3 Generation and analysis of the simulated data set. Click here for file

Additional data file 4 Analysis of experimental data available at the project web site [35]. Click here for file
