# Supplementary material for: Segmentation-based detection of allelic imbalance and loss-of-heterozygosity in cancer cells using whole genome SNP arrays
Source: Genome Biol. 2008 Sep 16;9(9):R136. doi: 10.1186/gb-2008-9-9-r136 (PMC2592714; doi:10.1186/gb-2008-9-9-r136)

Additional data file 1

Figure S1
Triplet filtering for removal of non-informative homozygous SNPs after applying a fixed mBAF threshold. (**a**) Principle of triplet filtering. (**b**) Histogram of triplet sums for SNPs homozygous in the matched blood and not removed by the used mBAF threshold of 0.97 for 15 urothelial carcinomas (data set 2). Vertical red dashed line corresponds to a triplet sum of 0.8. (**c**) Boxplot of triplet sums for SNPs homozygous in the matched blood and not removed by mBAF cut-off 0.97 for 15 urothelial carcinomas. Vertical red dashed line corresponds to a triplet sum of 0.8. (**d**) mBAF profile of chromosome 8 in breast tumor 2 (data set 1) after removal of SNPs with mBAF > 0.97. SNPs colored in red have triplet sums > 0.8. (**e**) Triplet sums for SNPs on chromosome 8 in breast tumor 2 after removal of SNPs with mBAF > 0.97. SNPs colored in red have triplet sums > 0.8. (**f**) mBAF profile of chromosome 8 in breast tumor 2 after removal of SNPs with mBAF > 0.97 and SNPs with triplet sums > 0.8.

Figure S2
Comparison of sensitivity for detecting ten simulated allelic imbalances for five methods. SNPs called as AB in NA06991 were used to estimate the sensitivity for the methods in detecting allelic imbalances in the simulated data set with increasing normal cell contamination. Sensitivity was calculated for each method based on the number of correct calls of the type of allelic imbalance for each region (gain is identified as gain). Lines correspond to sensitivity for PennCNV (black), QuantiSNP (green), unpaired segmentation (red), paired segmentation (orange), and SOMATICs (blue).

Figure S3
Difference in technical variation between Affymetrix SNP arrays and Illumina BeadChips for an urothelial carcinoma. Urothelial carcinoma UC199_I (data set 2) was hybridized on an Illumina 370k BeadChip and an Affymetrix 250k Nsp Mapping array. (**a**) Illumina BAF estimates for chromosome 9. (**b**) Affymetrix BAF estimates for chromosome 9. (**c**) Illumina BAF estimates for chromosome 9 after removal of SNPs homozygous in the matched normal blood. (**d**) Affymetrix BAF estimates for chromosome 9 after removal of SNPs homozygous in the matched normal blood.

Figure S4
Modifications of the segmentation strategy to better handle Affymetrix WGG data. Urothelial tumor UC3 (data set 2) was hybridized on an Affymetrix 250k Nsp Mapping array. Red line represents the CBS segmentation profile. Chromosome 5 is displayed. Horizontal dashed lines indicate positions of 0.97 and 0.9 in mBAF. (**a**) Identical parameters as for Illumina data. (**b**) mBAF cut-off lowered to 0.9, triplet filtering lowered to 0.6 and median used for estimation of the segmented value in the CBS algorithm.

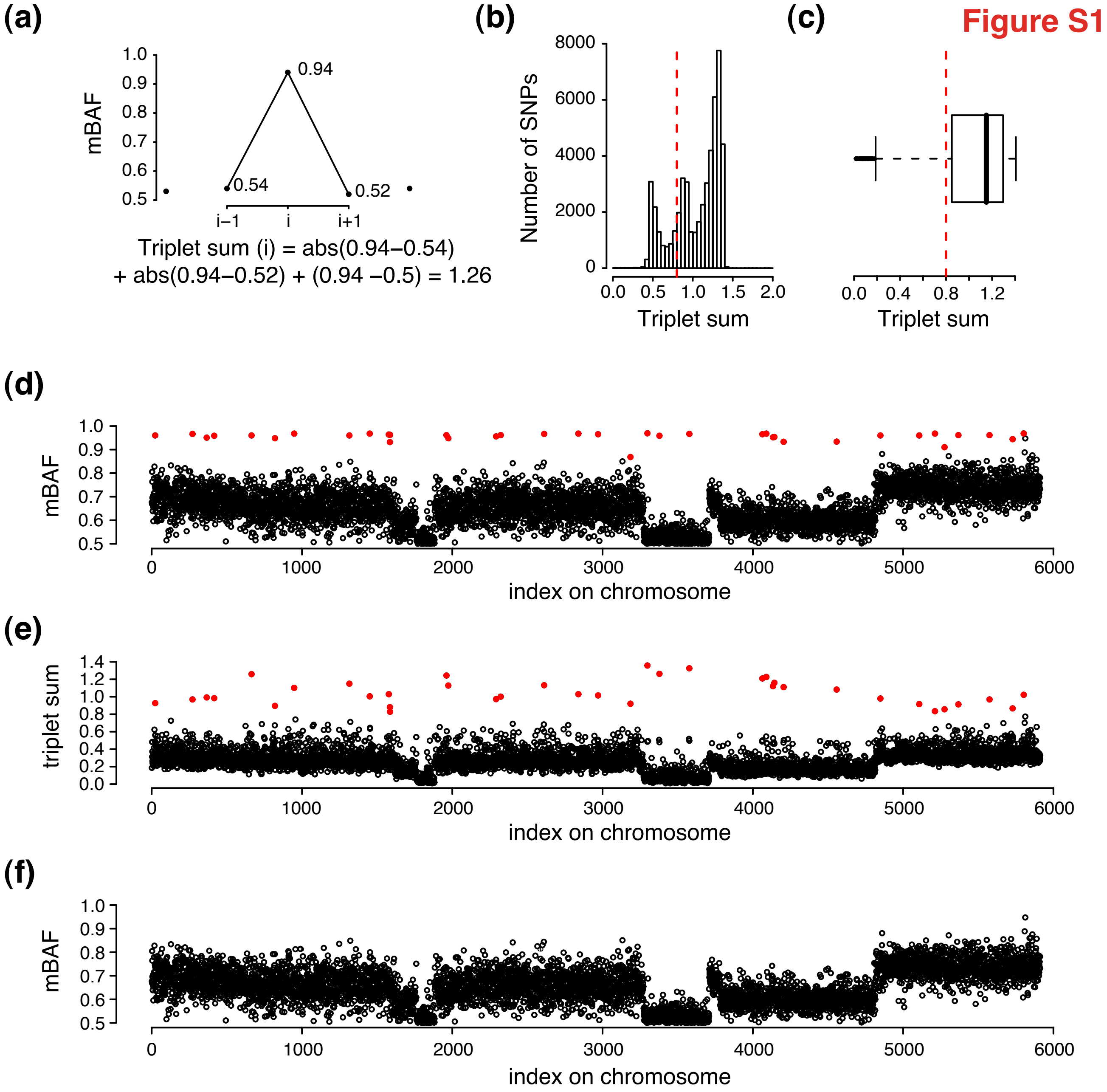
Figure S1
(a)
mBAF
0.94
0.54
0.52
i−1
i
i+1
Triplet sum (i) = abs(0.94−0.54)
+ abs(0.94−0.52) + (0.94 −0.5) = 1.26
(b)
Number of SNPs
Triplet sum
(c)
Triplet sum
(d)
mBAF
index on chromosome
(e)
triplet sum
index on chromosome
(f)
mBAF
index on chromosome

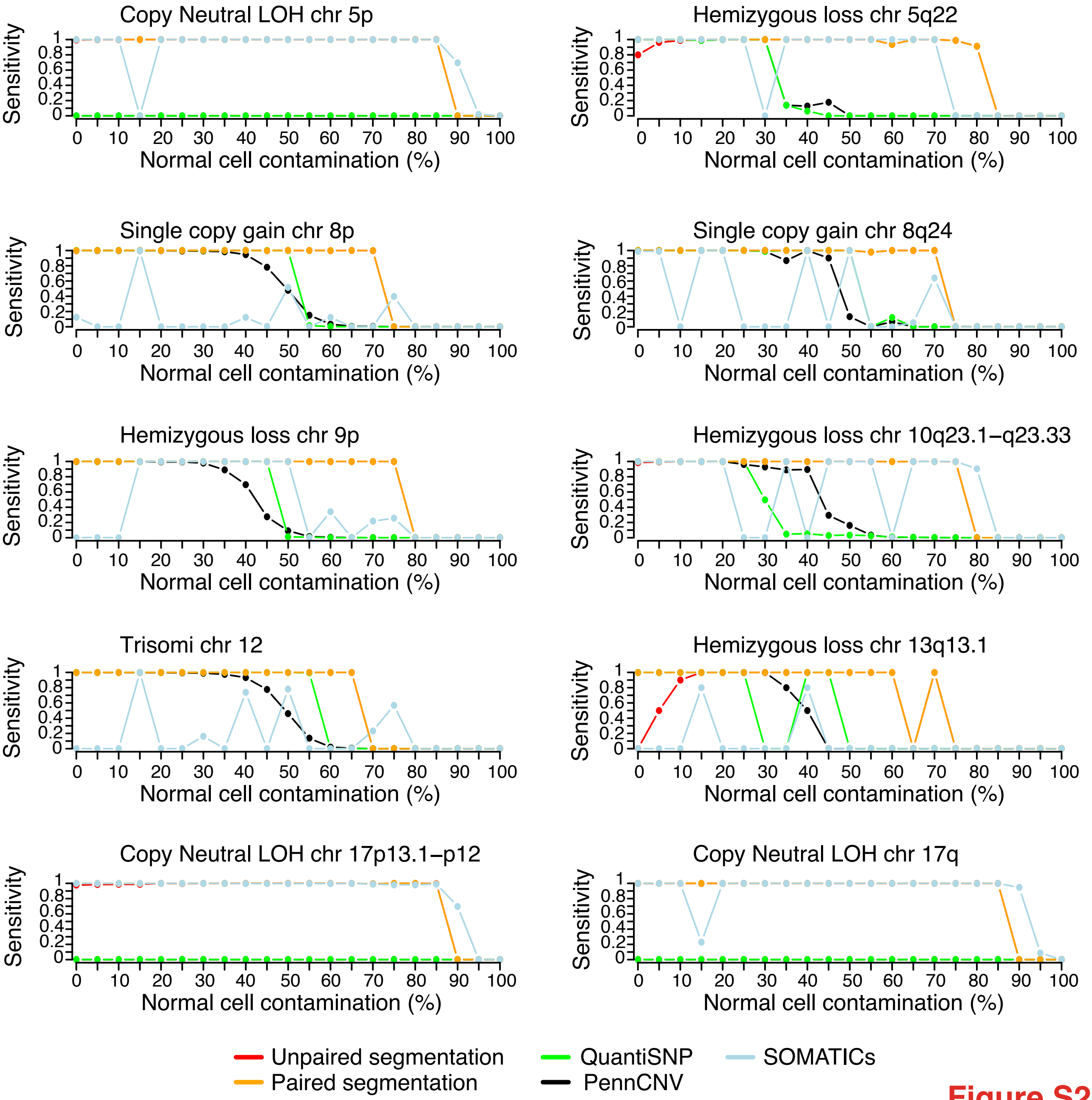

Copy Neutral LOH chr 5p
Hemizygous loss chr 5q22
Single copy gain chr 8p
Single copy gain chr 8q24
Hemizygous loss chr 9p
Hemizygous loss chr 10q23.1−q23.33
Trisomi chr 12
Hemizygous loss chr 13q13.1
Copy Neutral LOH chr 17p13.1−p12
Copy Neutral LOH chr 17q
Sensitivity
0 0.2 0.4 0.6 0.8 1
Normal cell contamination (%)
0 10 20 30 40 50 60 70 80 90 100
Unpaired segmentation
Paired segmentation
QuantiSNP
PennCNV
SOMATICs

Figure S2

Figure S3

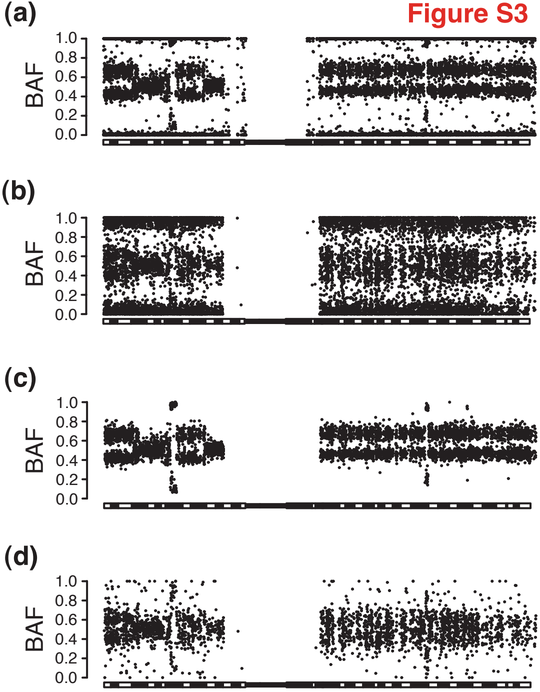

(a)
1.0
0.8
0.6
0.4
0.2
0.0
BAF
(b)
1.0
0.8
0.6
0.4
0.2
0.0
BAF
(c)
1.0
0.8
0.6
0.4
0.2
0.0
BAF
(d)
1.0
0.8
0.6
0.4
0.2
0.0
BAF

Figure S4

(a)

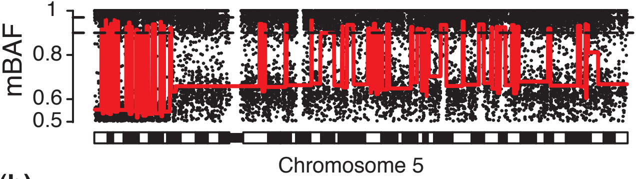

(b)

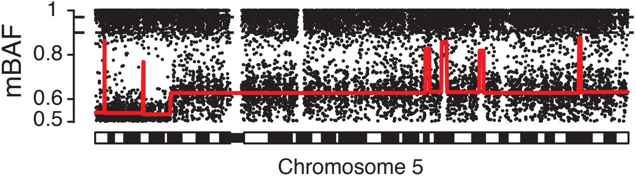

Supplement: Additional data file 1 — Implementation of the segmentation approach and analysis using the approach. [file gb-2008-9-9-r136-S1.pdf]
