# Supplementary material for: Segmentation-based detection of allelic imbalance and loss-of-heterozygosity in cancer cells using whole genome SNP arrays
Source: Genome Biol. 2008 Sep 16;9(9):R136. doi: 10.1186/gb-2008-9-9-r136 (PMC2592714; doi:10.1186/gb-2008-9-9-r136)
Supplement: Additional data file 2 — Results using triplet filtering. [file gb-2008-9-9-r136-S2.pdf]

Additional data file 2

Effect of triplet filtering for removal of non-informative homozygous SNPs after fixed mBAF threshold.

Sample *	Nbr homozygous SNPs in matched blood	Nbr homozygous SNPs not removed by mBAF threshold **	Nbr homozygous SNPs not removed by mBAF threshold and triplet filter ***
UC199 I	220397	2118	434
UC152 I	221611	3235	1420
UC196 I	220382	2025	176
UC513 M1	221728	5259	884
UC295 R1 M3	220810	4038	870
UC354 R1 M3	224880	1878	784
UC395 I M2	221839	2925	191
UC200 I	221000	3691	920
UC211 R	221008	2527	444
UC9 I	221506	4319	1041
UC77 R1	221846	3553	375
UC288 R	222248	3717	732
UC456 R	221122	3012	1141
UC542 I	221761	5191	686
UC544 I	221171	3919	1047

* :Data set 2.

** : Nbr of homozygous SNPs in the matched blood remaining in the tumor profile after application of mBAF threshold = 0.97

*** : Nbr of homozygous SNPs in the matched blood remaining in the tumor profile after application of mBAF threshold = 0.97 and triplet filter threshold = 0.8.
