# Supplementary material for: Segmentation-based detection of allelic imbalance and loss-of-heterozygosity in cancer cells using whole genome SNP arrays
Source: Genome Biol. 2008 Sep 16;9(9):R136. doi: 10.1186/gb-2008-9-9-r136 (PMC2592714; doi:10.1186/gb-2008-9-9-r136)
Supplement: Additional data file 3 — Generation and analysis of the simulated data set. [file gb-2008-9-9-r136-S3.pdf]

Additional data file 3

Construction of simulated data sets

Overview
A simulated data set was created to investigate the sensitivity and specificity of the segmentation approach compared to PennCNV [12], QuantiSNP [13] and SOMATICs [17]. The simulated data set was based on the diploid HapMap sample NA06991 hybridized on an Illumina HumanHap550 Genotyping BeadChip. NA06991 is part of the Illumina reference genotype cluster for the HumanHap550 Genotyping BeadChips. Different types of allelic imbalances were added to NA06991 at distinct genomic locations using the equations for the theoretical mBAF levels of single copy gain, hemizygous loss and copy neutral loss of heterozygosity (LOH). The simulated data set consist of 21 versions of the modified NA06991 sample with varying degree of normal cell contamination, starting from 0 % up to 100% in 5% increments.

Equations for allelic imbalances in diploid genomes
Since B allele frequency (BAF) represents an estimate of the proportion of allele A and B, equations for the theoretical value of different types of allelic imbalances in diploid samples can readily be formed. BAF data is expected to be symmetrical around 0.5 when the distribution of AA, AB and BB genotypes are roughly equal in a selected SNP probe set. Transforming BAF into mirrored BAF (mBAF) by a reflection along the BAF = 0.5 axes removes the association between a SNP and an allele. A region of hemizygous loss can for a diploid genome now be estimated to have mBAF = 1/(1+c), where c is the fraction of cells not showing the allelic imbalance, e.g., contaminating normal cells. Similarly, a copy neutral event can be estimated to have mBAF = (2-c)/2 and a single copy gain to have mBAF = (2-c)/(3-c).

Modeling of variation in BAF and copy number estimates for introduced allelic imbalances
To mimic a tumor sample a number of allelic imbalances were added to NA06991 (Table 1). The variation in BAF for the introduced allelic imbalances were set to the same standard deviation as for heterozygous SNPs in NA06991 = 0.038. BAF estimates for the different allelic imbalances were next modeled using a normal distribution with mean equaling the theoretical value for the type of imbalance given by the equations above and standard deviation = 0.038. For copy number estimates the data from X chromosome experiments in Peiffer et al. 2006 [6] were used to model the decrease of copy number estimates by increasing fraction of normal cell contamination. A linear model was fit to the three points corresponding to hemizygous loss, 2n and single copy gain in [6]. The resulting equation (y= 0.4735x – 1.0017) was split into one part modeling increase in copy number by normal cell contamination for hemizygous loss:

```
If( 1≤ x < 2){
        Copy number = 0.4735x – 1.0017                    [Eq 1]
}
```

and one part modeling decrease in copy number by normal cell contamination for single copy gain:

If (2<x ≤ 3){

$$\text{Copy number} = 0.4735x - 1.0017 \qquad \text{[Eq 2]}$$

}

With a normal cell contamination of 50%, x translates to 0.5*2+0.5*1=1.5 for equation 1 (hemizygous loss) and 0.5*2+0.5*3 = 2.5 for equation 2 (single copy gain). Normal copy number, 2N, was sampled from a normal distribution N(0.0141, 0.225) based on the experimental data for NA06991. The same standard deviation was used for regions of copy number loss and mono allelic gain. Average standard deviation for copy number estimates is 0.209 in the HumanHap 550 reference genotype cluster. Average standard deviation for heterozygous BAF estimates is 0.045 in the HumanHap 550 reference genotype cluster.

The introduced allelic imbalances are described in table 1. Due to the random sampling for copy number 2N, no true copy number variation (CNV) regions are expected. For BAF data, only heterozygous SNPs in NA06991 were modeled to the theoretical values for the different allelic imbalances. For copy number estimates, all SNPs within the defined genomic regions were remodeled according to the linear regression fit and type of copy number alteration.

Table 1. Introduced allelic imbalances in NA06991.

Aberration ID	Region	Base pairs	Nbr of BAF modified heterozygous SNPs
1	Hemizygous loss 9p	Chr9:1-50600000	3889
2	Hemizygous loss 10q23.1-q23.33	chr10:84504379-94825178	648
3	126kb hemizygous loss	chr13:31766569-31892852	10
4	731kb hemizygous loss	chr5:111789971-112521346	79
5	Mono allelic gain 8p	chr8:1-45200000	3830
6	Trisomi chr 12	chr12:1-132449811	8818
7	775kb mono allelic gain	chr8:128432670-129207869	91
8	Copy neutral LOH 5p	chr5:1-47700000	2756
9	Copy neutral LOH chr 17q	chr17:22800000-78774742	3205
10	4.3 Mb Copy neutral LOH	chr17:7431864-11747138	308
Total number of BAF modified heterozygous SNPs in NA06991			23634
Total nbr SNPs chromosome 1-22 in NA06991			547359
Total nbr of heterozygous SNPs in NA06991			176207
