# Supplementary material for: Segmentation-based detection of allelic imbalance and loss-of-heterozygosity in cancer cells using whole genome SNP arrays
Source: Genome Biol. 2008 Sep 16;9(9):R136. doi: 10.1186/gb-2008-9-9-r136 (PMC2592714; doi:10.1186/gb-2008-9-9-r136)
Supplement: Additional data file 4 — Analysis of experimental data available at the project web site [35]. [file gb-2008-9-9-r136-S4.pdf]

Additional data file 4

Supplemental data consisting of chromosome plots of the simulated data set (21 samples), 7 paired tumors hybridized on Illumina 300K and 370K BeadChips, and 2 urothelial tumors hybridized on Affymetrix 250K Nsp arrays are available at the project webpage: http://baseplugins.thep.lu.se/wiki/se.lu.onk.BAFsegmentation.

For the simulated Illumina data, each tumor sample was analyzed with PennCNV, QuantiSNP, SOMATICs and unpaired segmentation. For each tumor sample, B allele frequency estimates and log R ratios are shown together with regions called as allelic imbalance (gain, loss or copy neutral) for the different methods. Bars indicate allelic imbalances detected by (in order from top to bottom) unpaired segmentation (red), SOMATICs (blue), PennCNV (black) and QuantiSNP (green). SNPs homozygous in NA06991 have been removed from the B allele frequency plots to make the introduced tumor specific allelic imbalances more clear. Only chromosomes with simulated allelic imbalances are shown. Horizontal dashed lines in the B allele frequency plots correspond to values of 0.03, 0.1, 0.5, 0.9, and 0.97.

For experimental Illumina hybridizations, each tumor sample was analyzed with PennCNV, QuantiSNP, SOMATICs, dChipSNP and unpaired segmentation. For each tumor sample B allele frequency estimates and log R ratios are shown together with regions called as allelic imbalance (gain, loss or copy neutral) for the different methods. Bars indicate allelic imbalances detected by (in order from top to bottom) unpaired segmentation (red), SOMATICs (blue), PennCNV (black), QuantiSNP (green) and dChipSNP (brown). SNPs homozygous in the matched normal sample have been removed from the B allele frequency plots to make the tumor specific allelic imbalances more clear. Horizontal dashed lines in the B allele frequency plots correspond to values of 0.03, 0.1, 0.5, 0.9, and 0.97.

For Affymetrix hybridizations, each tumor sample was analyzed with CNAG version 2, dChipSNP and unpaired segmentation. For each tumor sample B allele frequency estimates and log R ratios are shown together with regions called as allelic imbalance (gain, loss or copy neutral) for each method. Bars indicate allelic imbalances detected by (in order from top to bottom) unpaired segmentation (red), CNAG (blue) and dChipSNP (brown).
